# A real-time, practical sensor fault-tolerant module for robust EMG pattern recognition

**DOI:** 10.1186/s12984-015-0011-y

**Published:** 2015-02-19

**Authors:** Xiaorong Zhang, He Huang

**Affiliations:** School of Engineering, San Francisco State University, 1600 Holloway Ave, San Francisco, CA USA; NCSU/UNC Rehabilitation Engineering Center (REC), NCSU/UNC Department of Biomedical Engineering, North Carolina State University, 4402C Engineering Building III, Raleigh, NC USA; University of North Carolina at Chapel Hill, 150A MacNider Hall, Chapel Hill, NC USA

**Keywords:** Sensor fault-tolerant, EMG pattern recognition, Real-time, Signal disturbance, Practical, Robust, Outlier detection, Self-recovery

## Abstract

**Background:**

Unreliability of surface EMG recordings over time is a challenge for applying the EMG pattern recognition (PR)-controlled prostheses in clinical practice. Our previous study proposed a sensor fault-tolerant module (SFTM) by utilizing redundant information in multiple EMG signals. The SFTM consists of multiple sensor fault detectors and a self-recovery mechanism that can identify anomaly in EMG signals and remove the recordings of the disturbed signals from the input of the pattern classifier to recover the PR performance. While the proposed SFTM has shown great promise, the previous design is impractical. A practical SFTM has to be fast enough, lightweight, automatic, and robust under different conditions with or without disturbances.

**Methods:**

This paper presented a real-time, practical SFTM towards robust EMG PR. A novel fast LDA retraining algorithm and a fully automatic sensor fault detector based on outlier detection were developed, which allowed the SFTM to promptly detect disturbances and recover the PR performance immediately. These components of SFTM were then integrated with the EMG PR module and tested on five able-bodied subjects and a transradial amputee in real-time for classifying multiple hand and wrist motions under different conditions with different disturbance types and levels.

**Results:**

The proposed fast LDA retraining algorithm significantly shortened the retraining time from nearly 1 s to less than 4 ms when tested on the embedded system prototype, which demonstrated the feasibility of a nearly “zero-delay” SFTM that is imperceptible to the users. The results of the real-time tests suggested that the SFTM was able to handle different types of disturbances investigated in this study and significantly improve the classification performance when one or multiple EMG signals were disturbed. In addition, the SFTM could also maintain the system’s classification performance when there was no disturbance.

**Conclusions:**

This paper presented a real-time, lightweight, and automatic SFTM, which paved the way for reliable and robust EMG PR for prosthesis control.

## Background

Electromyographic signal (EMG) pattern recognition (PR) is a widely used method for classifying user intent for neural control of artificial limbs [1-6]. However, unreliability of surface EMG recordings over time is a challenge for applying the EMG PR-controlled prostheses in clinical practice. Disturbances in EMG recordings are generally unpredictable in time, type, and level. Movement artifacts, environmental noises, electrode location shifts, loose electrode-skin contacts, user fatigue, and other conditions may all cause changes in the EMG characteristics and thus lead to inaccurate identification of user intent and threaten the prosthesis control reliability [7-11].

In order to make EMG PR clinically viable for control of artificial limbs, several strategies have been developed to address the challenge of unreliability in EMG recordings. Hargrove et al. [12], Young et al. [13,14], and Muceli [15] suggested several methods for making EMG PR-controlled systems more robust to electrode shift, including employing a new EMG PR training method [12], investigating the effects of electrode size and orientation [13], changing interelectrode distance and electrode configuration [14], and extracting control signals by linear factorization of multi-channel EMG recordings [15]. Sensinger et al. [9], Tommasi et al. [16], and Chen et al. [17] developed adaptive learning schemes to deal with variations in EMG signals for reliable EMG pattern classification. Lopez et al. [18] proposed a robust EMG sensing system by fusing redundant information of EMG signals to reduce the sensitivity of the control system relative to electrode failures. Tkach et al. [19] suggested several time-domain features that were resilient to EMG signal change caused by muscle fatigue and exerted force levels. Hahne et al. [20] employed a spatial filter in high-density EMG signal processing to obtain robustness to sensor noise. Geng et al. [21] developed a two-stage cascade classifier with the first classifier for limb position identification and the second for limb motion classification to reduce the effect of limb position variation on classification performance. Simon et al. [22] implemented a decision-based velocity ramp as a postprocessing step for the EMG PR algorithm to diminish the effect of misclassifications on the prosthesis movement. Amsuss et al. [23] proposed a self-correcting EMG PR-controlled system by adding a postprocessing algorithm to the existing EMG PR algorithm to detect and remove misclassifications of the system.

Our previous study also proposed a robust EMG PR interface for locomotion modes recognition based on the concept of sensor fault tolerance [24]. The proposed sensor fault-tolerant module (SFTM) consists of multiple sensor fault detectors and a self-recovery mechanism. The signals recorded from redundant EMG sensors are monitored by sensor fault detectors. The self-recovery mechanism will remove the EMG signals detected as abnormal recordings from the input of the pattern classifier. The simulation results showed that the SFTM maintained classification performance when one out of ten signals recorded from gluteal and thigh muscles was distorted and recovered about 20% of classification accuracy when four signals were distorted simultaneously.

While our previous study has demonstrated a promising concept of our design, the SFTM was evaluated offline on simulated constant disturbances. In order to implement this concept in real-time and apply the developed system in practice, three major requirements must be satisfied.

First, the self-recovery strategy may require frequent retraining of the pattern classifier. The previous training procedure is computationally demanding, which involves reorganization of the feature matrices extracted from the original training data, computation of the new parameters for the retrained classifier, and reorganization of the input data that is to be sent to the new classifier for motion classification. Whether or not this retraining procedure can be completed sufficiently fast in real time, especially when frequent retraining occurs, is the key to the success of applying SFTM to improve the robustness of EMG PR.

Second, the previous designs of the sensor fault detector are impractical [24-26]. They either require the models of EMG signals under different types of disturbances, which are difficult to establish because the disturbances could be diverse and hard to predict in the real world [24], or need manual parameter adjustment to handle various disturbance types [25,26]. A practical sensor fault detector is required in the proposed SFTM, which should be adaptive to various potential disturbances and is practical to build. Prompt detection is necessary in order for the disturbances to be addressed before they impair the performance of EMG PR. In addition, since disturbances do not always exist in the real world, the sensor fault detection should not harm the performance of EMG PR when there is no disturbance.

Last, to apply the designed robust EMG PR interface in practice, the SFTM needs to be seamlessly integrated with the PR module as a real-time system. The SFTM has to be lightweight in terms of computation and memory overhead, especially when implemented on embedded systems for prosthesis control, because embedded computers are usually resource constrained and typically have processors with relatively slow system clock and limited memory.

In this study, we aimed to develop a real-time, practical EMG PR interface with SFTM for artificial arms. A novel fast and efficient retraining algorithm based on linear discriminant analysis (LDA) was developed. The proposed retraining method had been preliminarily evaluated in our previous study to validate its feasibility for real-time processing [27]. A simple, fully automatic outlier detector was designed for prompt sensor fault detection. The detector was built from normal training data only and was not restricted to any type of disturbance. A new method for tuning the outlier threshold was developed, in which the detector parameters are calculated in the training procedure automatically. No more tuning step is required in the testing phase. The proposed retraining strategy and the sensor fault detector were designed to make the most efficient use of the existing information obtained from EMG PR, and thus to minimize the overhead of the SFTM to the EMG PR interface. These components of SFTM were then integrated with the EMG PR module and tested on five able-bodied subjects and a transradial amputee in real-time for classifying multiple hand and wrist motions under different conditions with or without disturbances. The experimental results demonstrated the feasibility of a practical SFTM for robust and reliable EMG PR for artificial arm control.

## Methods

### Architecture of the robust EMG PR interface

**Figure 1:**
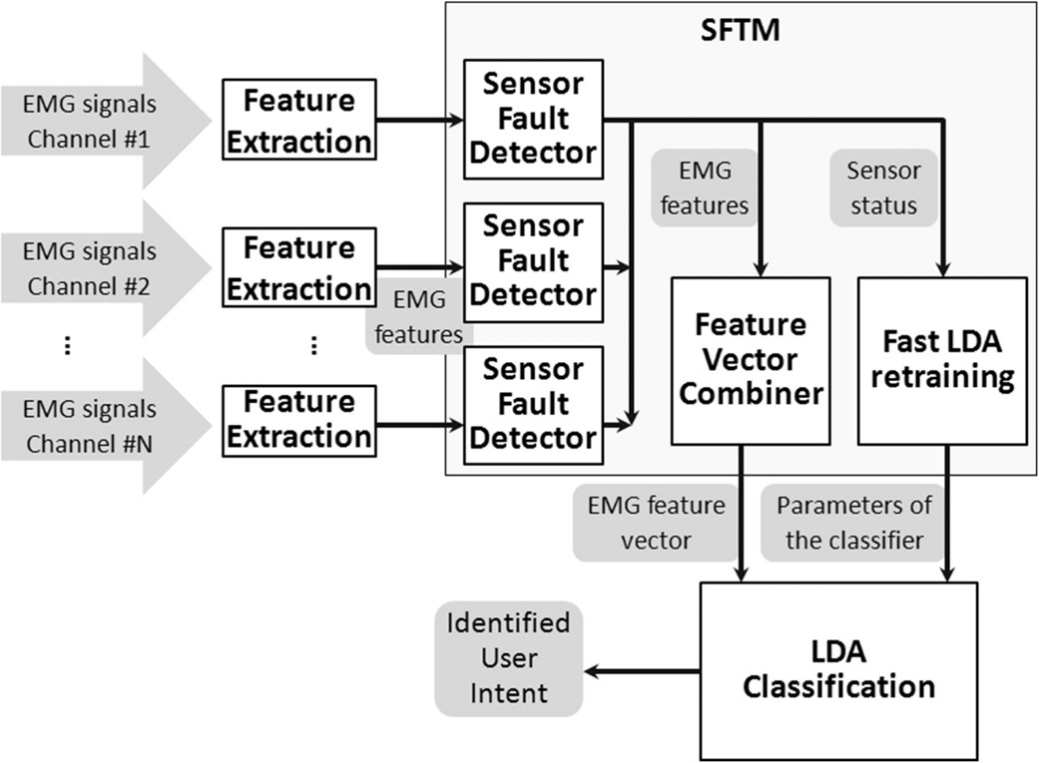
**Block diagram of the system architecture of the robust EMG PR interface.** The white blocks represent the system components and the gray shapes represent the information passed on between the system components. SFTM = sensor fault-tolerant module. LDA = linear discriminant analysis.

Figure 1 shows the overall structure of the robust EMG PR interface, which seamlessly integrates the EMG PR module with the SFTM. The system inputs are multiple channels of EMG signals. The input signals are preprocessed and segmented by overlapped sliding analysis windows. In every window, four time-domain (TD) features (mean absolute value, number of zero crossings, waveform length, and number of slope sign changes [1]) of the EMG signals are extracted from each input channel and then fed into the self-recovery module. The SFTM mainly consists of three components: multiple *sensor fault detectors*, a *feature vector combiner*, and a *fast LDA retraining procedure*. The sensor fault detectors closely monitor the features of individual EMG signals to detect various disturbances. Based on the detection results, the feature vector combiner concatenates the EMG features extracted from ‘normal’ channels into a feature vector as the input for pattern classification. If no disturbance is detected, the feature vector will be directly sent to the classifier generated from the original training data. If one or more signals are determined as ‘abnormal’, the features of the distorted signals will not be included in the feature vector and the original classifier will be inapplicable. In this case, the fast LDA retraining procedure will be triggered and a new classifier will be generated. Then the feature vector, composed of features extracted from ‘normal’ signals, will be fed into the new classifier for PR.

### Fast LDA-based retraining algorithm

The response time of the retraining algorithm is very critical to the design of the real-time SFTM. Linear discriminant analysis (LDA) is a widely used method for EMG PR because of its comparable classification accuracy to more complex classifiers and the computational efficiency for real-time processing [1,3,25]. After examining the details of the LDA algorithm, we developed a fast and memory efficient LDA retraining algorithm by making the most efficient use of existing information.

The principle of the LDA-based PR strategy is to find a linear combination of features which separates multiple classes *C*_*g*_ (*g* ∈ [1, *G*]). Here *G* denotes the total number of studied classes. Suppose $\overline{f}$ is the feature vector of one analysis window, *μ*_*g*_ is the mean vector of class *C*_*g*_ and every class shares a common covariance matrix ∑, the linear discriminant function is defined as

$$ {d}_{C_g}={\overline{f}}^T{\varSigma}^{-1}{\mu}_g-\frac{1}{2}{\mu_g}^T{\varSigma}^{-1}{\mu}_g. $$

During the training procedure, ∑ and *μ*_*g*_ are estimated based on the feature matrix calculated from the training data. The estimations of ∑ and *μ*_*g*_ are expressed as

$$ \tilde{\varSigma}=\frac{1}{G}{\displaystyle \sum_{g=1}^G\frac{1}{K_g-1}\left({F}_g-{M}_g\right){\left({F}_g-{M}_g\right)}^T} $$

and

$$ {\tilde{\mu}}_g=\frac{1}{K_g}{\displaystyle \sum_{k=1}^{K_g}{\overline{f}}_{C_g,k}} $$

where *K*_*g*_ is the number of analysis windows in class *C*_*g*_; ${\overline{f}}_{C_g,k}$ is the *k*_*th*_ observed feature vector in class *C*_*g*_; ${F}_g=\left[{\overline{f}}_{C_g,1},{\overline{f}}_{C_g,2},\dots, {\overline{f}}_{C_g,k},\dots, {\overline{f}}_{C_g,{K}_g}\right]$ is the feature matrix of class ${C}_g;\;{\overline{f}}_{C_g,1},{\overline{f}}_{C_g,2},\dots, {\overline{f}}_{C_g,k},\dots, {\overline{f}}_{C_g,{K}_g}$ is the mean matrix which has the same dimension as *F*_*g*_. In a feature vector ${\overline{f}}_{C_g,k}={\left[{f_1}^T,{f_2}^T,\dots, {f_n}^T,\dots, {f_N}^T\right]}^T,\;N$ is the total number of EMG input channels and *f*_*n*_ denotes the four EMG features extracted from the *n*_*th*_ channel.

In the previous retraining strategy [24], after the initial training process is done, the original EMG feature matrices *F*_*g*_ (*g* ∈ [1, *G*]) are stored in the memory for later use in the retraining process. During the retraining procedure, for each class, a new EMG feature matrix *F*_*g*_ ' is reorganized by removing the feature rows corresponding to the disturbed channels from *F*_*g*_. The mean vector of each class ${\tilde{\mu}}_g\hbox{'}$ and the new common covariance matrix $\tilde{\varSigma}\hbox{'}$ are then recalculated based on *F*_*g*_ '. Our experimental analysis has shown that the calculation of $\tilde{\varSigma}\hbox{'}$ is the most computational intensive task in the retraining procedure, which accounts for more than 90% of the total processing time. This is because for each class, a large amount of analysis windows are collected as the training data. The number of columns in *F*_*g*_ ' may vary from several hundreds to a few thousands, which leads to intensive numerical operations in calculating $\tilde{\varSigma}\hbox{'}$.

**Figure 2:**
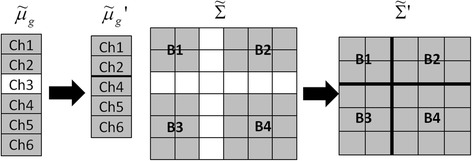
**An example of retrieving** $\tilde{\boldsymbol{\Sigma}}\hbox{'}$ **and** ${\tilde{\boldsymbol{\upmu}}}_{\mathbf{g}}\hbox{'}$ **from** $\tilde{\boldsymbol{\Sigma}}$ **and** ${\tilde{\boldsymbol{\upmu}}}_{\mathbf{g}}$. The example shows the process of retrieving $\tilde{\varSigma}\hbox{'}$ and ${\tilde{\mu}}_g\hbox{'}$ from $\tilde{\varSigma}$ and ${\tilde{\mu}}_g$ when a single EMG channel *Ch*3 is disturbed. The white blocks represent the elements associated with the disturbed channel.

In our new fast retraining strategy, the calculation of $\tilde{\varSigma}\hbox{'}$ and ${\tilde{\mu}}_g\hbox{'}$ can be skillfully avoided. The trick is, instead of the large feature matrices *F*_*g*_, only the mean vector ${\tilde{\mu}}_g$ and the common covariance matrix $\tilde{\varSigma}$ are stored in the memory after the initial training process is finished. Figure 2 shows an example of retrieving $\tilde{\varSigma}\hbox{'}$ and ${\tilde{\mu}}_g\hbox{'}$ from $\tilde{\varSigma}$ and ${\tilde{\mu}}_g$ when totally 6 EMG channels are in the system and a single EMG channel *Ch*3 is identified as ‘abnormal’. The white blocks represent the elements associated with the disturbed channel. ${\tilde{\mu}}_g\hbox{'}$ can be obtained by taking off the elements that are associated with the disturbed EMG channel from ${\tilde{\mu}}_g$. $\tilde{\varSigma}\hbox{'}$ is constructed by removing the corresponding rows and columns associated with the disturbed channel from $\tilde{\varSigma}$ and then merging the remaining four small matrices (*B*1, *B*2, *B*3, and *B*4 in Figure 2). If multiple EMG signals are disturbed, $\tilde{\varSigma}\hbox{'}$ and ${\tilde{\mu}}_g\hbox{'}$ can be obtained by doing the retrieving process repeatedly. Compared with the previous retraining algorithm which requires intensive numerical operations and a large memory space, the new strategy dramatically accelerates the retraining speed and is much more memory efficient.

### Sensor fault detector

In this study, a simple, fully automatic outlier detection method based on Mahalanobis distance analysis [28] was designed for EMG sensor fault detection because of several reasons. First, the detector is built only from normal training data and can detect new types of intrusions [29], which is more practical than those who need prior knowledge of both normal data and all disturbance types. Second, the detector parameters can be calculated in the training procedure automatically. No more tuning step is required in the testing phase. Last, the detection algorithm is computationally efficient for real-time implementation. Mahalanobis distance analysis is closely related to LDA, thus some calculation results from the LDA classification can be directly reused in the outlier detection.

We assume that various types of disturbances are qualitatively different from EMG signals during normal motion activities. Since normal EMG signals of each motion class are already collected in the training session, for each class, a normal EMG model is established from the training data. If a new piece of testing data has a large deviation from all the normal EMG models, it will be flagged as a disturbance. In our proposed system, each individual EMG signal is monitored by an outlier detector. In every analysis window, the 4 × 1 feature vector *f*_*n*_ extracted from the *n*^th^ EMG signal is processed as a new instance. The Mahalanobis metric [28] is used to estimate the distances of the new instance from the normal model of each class. The instance is identified as

$$ \left\{\begin{array}{c}\hfill\ \mathrm{abnormal},\ \mathrm{if}\ \underset{g\in \left[1,G\right]}{ \min }{\left({f}_n-{\tilde{\mu}}_{g,n}\right)}^T{\tilde{\sum}}_{nn}^{-1}\left({f}_n-{\tilde{\mu}}_{g,n}\right)>{\tau}_n\hfill \\ {}\hfill\ \mathrm{normal},\kern1.25em \mathrm{if}\ \underset{g\in \left[1,G\right]}{ \min }{\left({f}_n-{\tilde{\mu}}_{g,n}\right)}^T{\tilde{\sum}}_{nn}^{-1}\left({f}_n-{\tilde{\mu}}_{g,n}\right)\le {\tau}_n\hfill \end{array}\right. $$

where for a specific channel $n,\;{\tilde{\mu}}_{g,n}$ is the estimated mean vector of class ${C}_g\ \left(g\in \left[1,G\right]\right),\;{\tilde{\varSigma}}_{nn}$ is the estimated 4 × 4 common covariance matrix for all classes. ${\tilde{\mu}}_{g,n}$ and ${\tilde{\varSigma}}_{nn}$ are calculated in the training phase, and can be easily extracted from the corresponding positions in ${\tilde{\mu}}_g$ and $\tilde{\varSigma},$ respectively. ${\left({f}_n-{\tilde{\mu}}_{g,n}\right)}^T{\tilde{\varSigma}}_{nn}^{-1}\left({f}_n-{\tilde{\mu}}_{g,n}\right)$ calculates the estimated squared Mahalanobis distance from the new instance *f*_*n*_ to the normal model of class *C*_*g*_ ; *τ*_*n*_ is the outlier threshold. The value of *τ*_*n*_ is chosen to reflect the sensitivity of the outlier detector. Choosing a smaller value of *τ*_*n*_ will result in a higher detection rate as well as a higher false alarm rate. While a higher detection rate allows the SFTM to identify abnormal signals and recover classification decisions more efficiently, a higher false alarm rate might result in more incorrect removals of important good EMG channels from the PR system and thus might lead to classification errors. In the real world, the occurring time, duration, and type of disturbance cannot always be well predicted. Because disturbance does not always occur, it is essential for the SFTM not to significantly harm the PR performance when there is no disturbance. Since all training data are considered as ‘normal’, in our designed sensor fault detector, the value of *τ*_*n*_ is automatically estimated in the training phase by specifying the value of tolerable classification accuracy degradation (TCAD). TCAD is defined as the allowed maximum accuracy degradation of EMG PR when the SFTM is applied on normal signals. In our experiments, the value of TCAD was set to be 0.2%.

### Participants

This study was conducted with Institutional Review Board (IRB) approval and informed consent of all subjects. Five able-bodied subjects (four males and one female; subjects AB1-AB5), free from orthopedic or neurological pathologies, and a congenital transradial (TR) amputee (male; subject TR1) were recruited. The average age of subjects AB1-AB5 is 28.6 ± 2.7 years. Subject TR1 is 31 years old and the length of his deficient left forearm is 9 cm from the lateral epicondyle to the distal end of bone. The subject has been using a two-site myoelectric prosthetic hand in his daily life.

### EMG data collection

For each able-bodied subject, seven different classes of motion (3 degrees of freedom and a rest class) were required to complete the test. These motions were no movement, wrist supination, wrist pronation, hand close, hand open, wrist flexion, and wrist extension. Six EMG signals were recorded from muscles on the subject’s dominant forearm. The monitored muscles included the *flexor carpi ulnaris* (FCU), *flexor carpi radialis* (FCR), *extensor carpi ulnaris* (ECU), *extensor carpi radialis* (ECR), *extensor pollicis longus* (EPL)*, and palmaris longus* (PL). The EMG electrodes were placed over the anatomical locations described in [30].

For the subject with the congenital limb deficiency (subject TR1), the motions chosen to elicit EMG signals were no movement, wrist supination, wrist pronation, hand close, and hand open, as determined by the ability of the subject. Six EMG electrodes were placed around his deficient forearm. Since PR control does not require independent muscle control sites [8,31], the electrode sites were determined by palpation while the subject performed the appropriate contractions. Wrist flexion and wrist extension were investigated on subject TR1 originally but were observed to be frequently mixed up with other motions. The subject was not able to consistently perform more than five distinct motions; therefore only five motion classes (2 degrees of freedom and a rest class) were chosen for subject TR1.

Electrode placement was prepared by shaving any excessive body hair in the site regions, and applying an alcohol prep pad. Disposable self-adhesive bipolar surface EMG electrodes (Norotrode, Myotronics Inc.) were used in this study. Each Norotrode was connected to an EMG pre-amplifier (MA-420-002, Motion Lab System Inc.), which band-pass filtered the EMG signals between 10 Hz and 3,500 Hz with a pass-band gain of 20. A single ground electrode was placed on the back of the elbow for all the subjects. All data were collected by a 16-channel EMG system (MA-300-16, Motion Lab System Inc), digitized by a data acquisition system with 16-channel 16-bit analog-to-digital converters (USB-1616HS, Measurement Computing Corporation), and sampled at 1000 Hz per channel. The sampled data were segmented into overlapped analysis windows with 160 ms length and 20 ms increment, resulting in a new decision every 20 ms [32].

### Disturbance types

To evaluate the performance of the proposed sensor fault detector and the self-recovery strategy, three common disturbances of EMG recordings have been investigated in this study: *contact artifacts*, *loose contacts*, and *baseline noise* [10,33]. Note that the proposed SFTM is not restricted to any specific type of disturbance. These are just three representative disturbance types that are used to demonstrate the feasibility of the SFTM. The following subsections briefly describe the data collection procedure of each type of disturbances.

#### Contact Artifact (CA)

**Figure 3:**
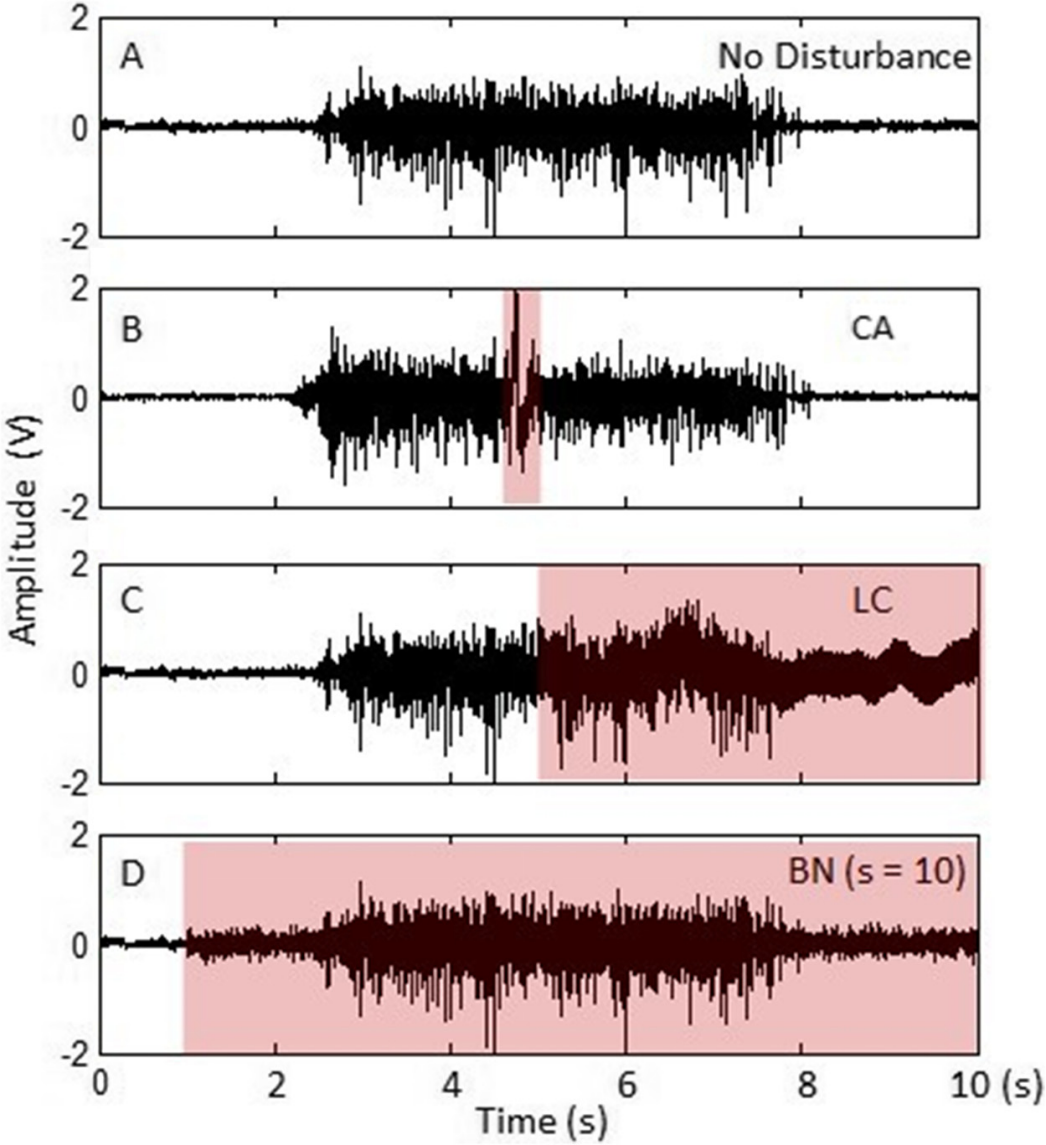
**Examples of EMG signals with different distortions. A**: normal EMG signal without disturbance; **B**: EMG signal contaminated by contact artifacts; **C**: EMG signal with loose contact condition; **D**: EMG signal with simulated baseline noise (*s* = 10). The red panels indicate the distorted EMG signals.

This is a common low-frequency noise that may lead to large drifts in EMG magnitude from baseline and is very difficult to totally remove [10]. Contact artifacts can be generated in many different situations in practice. In our experiments, we manually added contact artifacts to normal EMG signals by tapping one or multiple EMG electrodes during the subject’s normal motion activities because it is a simple yet controllable way of generating the disturbances in the lab environment [10]. Figure 3B shows an example of adding contact artifacts into a normal EMG signal.

#### Loose Contact (LC)

Loose electrode-skin contacts or contact lost can cause variations of electrode-skin impedance over time and thus lead to low-quality signals [33]. To evaluate our system’s ability to handle this type of disturbance in real-time experiments, it is impractical to abruptly loosen or peel off the electrodes from skin during the subject’s normal motion activities because it may interfere with the subject’s performance and even damage the electrodes. Instead, we simulated this situation by two steps: 1) we first gently peeled off the bipolar electrodes when the subject was in the rest state such that the electrodes were still partially attached to the skin. The signals of loose contacts from each channel were then collected and stored. 2) The collected disturbance data were fused with normal EMG signals in a way to mimic the randomness of real conditions where the disturbed channel, the timing, and duration of the disturbances are unpredictable. Assume in a testing trial, the number of disturbance segments to be added into a single EMG channel is *I* and the total number of EMG signals is *N*. The testing data (i.e. normal EMG with disturbances) was constructed as

2 $$ \left\{\begin{array}{c}\hfill {E}_n(t)+{D}_{n,i}(t),\kern1.5em t\_ star{t}_{n,i}<t<t\_en{d}_{n,i}\hfill \\ {}\hfill {E}_n(t),\kern6em \mathrm{otherwise}\kern1.5em \hfill \end{array}\right.,\;\left(1\le i\le I,\kern0.5em 1\le n\le N\right),\;\left(1\le i\le I,\kern0.5em 1\le n\le N\right), $$

where *E*_*n*_(*t*) denotes the normal EMG signal from the *n*^*th*^ channel; *D*_*n,i*_(*t*) denotes the data of the *i*^*th*^ disturbance segment to be added to the channel; *t* _ *start*_*n*,*i*_ and *t* _ *end*_*n*,*i*_ are the start and the end time of *D*_*n*,*i*_; *t* _ *end*_*n*,*i*_ − *t* _ *start*_*n*,*i*_ represents the length of the disturbance. The testing data construction was performed online to simulate the real-time conditions. Figure 3C displays an example of an EMG signal with the loose-contact condition.

#### Baseline Noise (BN)

**Figure 4:**
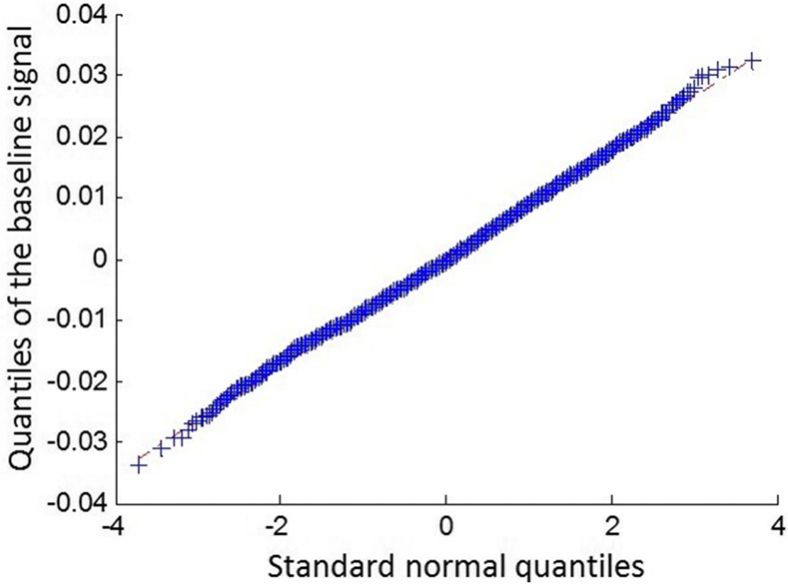
**The Q-Q plot of the quantiles of the baseline signal versus the quantiles of a standard normal distribution.** A Q-Q plot is a probability plot for comparing two probability distributions by plotting their quantiles against each other. The Q-Q plot shown in this figure approximately lies on a line, indicating that the distribution of the baseline noise and the Gaussian distribution are linearly related, so the baseline noise in this work was simulated as white Gaussian noise with different scales.

The baseline noise is composed of the thermal noise and the electrode-skin interface originated noise [10,18,34]. It can severely impair the resolution of signal recordings from surface electrodes. To generate the baseline noise, one trial of the baseline signal was first collected by asking the subject to completely relax the muscles for 20 s. To analyze the distribution of the baseline noise, the Quantile-Quantile (Q-Q) plot [35] of the quantiles of the collected baseline signal versus the quantiles of a standard normal distribution was investigated (Figure 4). The Q-Q plot approximately lies on a line as shown in Figure 4, indicating that the distribution of the baseline noise and the Gaussian distribution are linearly related, so we assume the baseline noise is white Gaussian noise with different scales. To simulate different levels of baseline noise, the standard deviation of the collected baseline signal *σ* was first estimated, and then the simulated baseline noise was generated by creating white Gaussian noise with a standard deviation as *s* ⋅ *σ*, where *s* could be used to adjust the scale of the baseline noise. The simulated baseline noise and the EMG signal were then fused in a similar way as described in (2). An example of an EMG signal with additional baseline noise (s = 10) is shown in Figure 3D.

### SFTM implementation on embedded system

To demonstrate the feasibility of realizing the proposed system on a real-time embedded system, a preliminary prototype of the robust EMG PR interface was implemented on the Gumstix Overo Air computer-on-module (COM), which contains a Texas Instruments OMAP3503 600 MHz processor based on the ARM Cortex-A8 architecture.

Classifier retraining used to be the most computational intensive part in the robust EMG PR interface. In this study, we compared two prototypes of the PR interface implemented on the Overo Air COM, one with the proposed fast LDA retraining algorithm, the other with the previous retraining strategy. By processing the same dataset, the computational time and the memory consumption of the two strategies were compared and discussed in the result section.

### Real-time experiments of the robust EMG PR interface on PC

The real-time experiments were conducted on a Matlab based implementation running on a laptop PC. The PC used was running 64-bit Windows 7 with processor at 3.1 GHz (Intel i5) and 4GB of RAM.

The experiments consisted of two sessions: training and testing. The training session was conducted first to collect training data for two purposes. One is to create the original motion classifier. The other is to build the sensor fault detector for each EMG channel. For each able-bodied subject, two trials of training data were collected. Each trial consisted of two repetitions of the following seven types of motion classes: no movement, wrist supination/pronation, hand close/open, and wrist flexion/extension. The subjects were not restrained in any way during data collection. All contractions were performed at a speed and strength determined by the subject, and they were encouraged to contract at a level that they felt comfortable to repeat for the duration of the experiment. Before the training trial started, the subjects were instructed to perform each motion task multiple times while the raw signals were viewed in real-time. The experimenter ensured that the signal quality of each EMG channel was good and that the gains of the amplifiers were appropriately set to avoid signal saturation. A Matlab implemented software GUI was developed to instruct the subject to elicit and relax contractions. During the training data collection, upon seeing a command of a motion class, the subject began the instructed contraction, and held the final position until seeing a command instructing them to relax the contraction. The duration of each motion was 4 s, and the rest periods were 8 s between successive contractions. There was a 3 s countdown display to cue the subject before switching to the next motion.

For the subject with the congenital limb deficiency, nine training trials were conducted, including two trials for each motion class (wrist supination, wrist pronation, hand close, and hand open), and one trial for the no movement class. Each data collection trial consisted of two repetitions of a single motion class (4 s duration) separated by the rest periods (8 s duration). The subject was instructed to simultaneously perform the same motion with his contra-lateral limb in order to aid him in visualizing the task. Sufficient rest time was allowed between successive trials to avoid muscle fatigue.

The collected training data were then processed in two pathways. First, the motion classifier was created by processing the data set using the LDA method. After the training process was done, the parameters of the generated classifier, the mean vector of each class ${\tilde{\mu}}_g$ and the common covariance matrix $\tilde{\varSigma}$ were saved in the memory for later use in the real-time testing session. Second, the same dataset was used to build the sensor fault detector for each EMG channel.

In the real-time testing session, each subject was required to perform the contractions under four different conditions with or without additional disturbances: 1) no disturbance (ND), 2) contact artifacts (CA), 3) loose contacts (LC), and 4) baseline noise (BN). For the BN disturbance, three different scales were tested with *s* configured as 5, 10, and 20, respectively. Each testing trial contains one repetition of all the investigated motion classes. The subjects performed all the contractions in a random order by following the instructions displayed on the software GUI. The duration of each contraction was 4 s and the rest period between successive contractions was 8 s. The disturbances were either manually introduced by the experimenter (CA) or fused with the collected EMG signals by software (LC and BN) in real-time. In the CA trials, the experimenter tapped 1–3 electrode(s) at a time during each contraction for 2–4 times. The experimenter was instructed to tap all the electrodes as evenly as possible during the testing trials so that each electrode could have nearly the same chance to be disturbed. For the LC and BN trials, one disturbance segment was added into each EMG channel for each contraction at a random start time. The duration of each disturbance was a random value within the range of 100–400 ms. For each subject, totally 30 testing trials were collected, 5 for each condition. To better evaluate the performance of the proposed self-recovery strategy, two types of classification decisions with and without the self-recovery module were compared in every analysis window. The software GUI displayed the status of each EMG sensor and the classification decisions in real-time. All the experiments were documented and videotaped for later analysis.

### Performance measurement

To evaluate the performance of the sensor fault detector, the detection rate (DR) and the false alarm rate (FAR) were computed. The metrics can be quantified as

$$ DR=\frac{{\displaystyle \sum_{n=1}^NT{P}_n}}{{\displaystyle \sum_{n=1}^N\left(T{P}_n+F{N}_n\right)}}\times 100\%, $$

and

$$ FAR=\frac{{\displaystyle \sum_{n=1}^N\left(F{P}_n\right)}}{{\displaystyle \sum_{n=1}^N\left(F{P}_n+T{N}_n\right)}}\times 100\%, $$

where *N* is the total number of EMG channels. For a single channel *n*, *TP*_*n*_, *FN*_*n*_, *FP*_*n*_, *TN*_*n*_ are the numbers of true positives, false negatives, false positives, and true negatives of the detector, respectively; *TP*_*n*_ + *FN*_*n*_ is the total number of disturbed observations, and *FP*_*n*_ + *TN*_*n*_ is the total number of normal observations. Note that for the testing trials disturbed by contact artifacts, the ground truth of the analysis windows was estimated according to the plots of the signals and the information recorded in the experiment documentations and videos, because tapping an electrode may affect multiple successive analysis windows and the exact starting times and durations of the disturbances were unknown.

In our experiments, since the false alarms were defined as the miss-identifications of the observations that did not belong to the manually introduced disturbances (i.e. CA, LC, and BN), it was interesting to observe that the false alarms not only caused classification errors occasionally, but also corrected some decision errors that might be caused by the inherent low quality of the EMG signal or other non-manually introduced disturbances. To evaluate the effect of false alarms on the classification decisions, two more metrics were calculated, including the ratio between the number of classification errors caused by false alarms and the total number of analysis windows affected by false alarms (EFAR), and the ratio between the number of classification errors corrected by false alarms and the total number of analysis windows affected by false alarms (CEFAR).

To evaluate the performance of the self-recovery strategy, the analysis windows in the testing trials were divided into four categories according to the total number of disturbed EMG channels (categorized as 0, 1, 2, and 3). The average classification accuracy (CA) of each category was calculated as

$$ CA=\kern0.5em \frac{\mathrm{Number}\ \mathrm{of}\ \mathrm{correct}\ \mathrm{classfication}\ \mathrm{decisions}\ \mathrm{in}\ \mathrm{the}\ \mathrm{category}}{\mathrm{Total}\ \mathrm{number}\ \mathrm{of}\ \mathrm{classification}\ \mathrm{decisions}\ \mathrm{in}\ \mathrm{the}\ \mathrm{category}}\kern0.5em \times \kern0.5em 100\%. $$

In addition, the classification accuracies of the system with and without the self-recovery module were both calculated and compared.

## Results

### Fast retraining algorithm vs. previous retraining algorithm

Tables 1 and 2 summarize the performance comparison between the proposed fast LDA retraining algorithm and the previous retraining algorithm running on the embedded system described in the section “SFTM Implementation on Embedded System”. Table 1 shows the processing time of the retraining algorithm when one, two, or three EMG channel(s) was/were disturbed. Table 2 compares the major memory consumption between our fast LDA retraining algorithm and the previous retraining algorithm. The original training data set contained EMG signals collected from six channels and seven classes (772 analysis windows for each class) for subjects AB1 – AB5, and six channels and five classes (772 analysis windows for each class) for subject TR1. It is shown that the new retraining algorithm was two orders of magnitude faster than the previous retraining strategy and meanwhile only consumed less than 1% of the memory usage of the old strategy. Furthermore, our proposed algorithm only took less than 4 ms to create the new classifier while the old strategy needed nearly 1 s, which is inadequate for real-time implementation. This result makes it possible for the new system to seamlessly extract EMG features, detect signal disturbances, retrain the classifier, perform PR, and produce the recovered classification decision in a sequence within the duration of one window increment (i.e. 20 ms in our experiments).

**Table 1:** **Processing time comparison between the new retraining method and the previous retraining method**

	**New fast retraining**	**Previous retraining**	**Speedup**
AB subjects (one channel removed)	3.78 ± 0.58 ms*	986.89 ± 49.71 ms*	267 ± 49
AB subjects (two channels removed)	2.93 ± 0.39 ms*	752.03 ± 25.71 ms*	260 ± 32
AB subjects (three channels removed)	2.21 ± 0.36 ms*	486.97 ± 21.11 ms*	242 ± 30
TR1 subject (one channel removed)	3.56 ms	711.21 ms	200
TR1 subject (two channels removed)	2.80 ms	476.56 ms	170
TR1 subject (three channels removed)	1.95 ms	307.02 ms	157

*Paired *t*-test on AB subjects denotes statistically significant difference between the processing time of the new retraining method and the previous method (p < 0.05).

**Table 2:** **Major memory usage comparison between the new retraining method and the previous retraining method**

	**New fast retraining**	**Previous retraining**
AB subjects (772 windows for each class, 7 classes, 6 channels, 4 features per channel)	${\tilde{\mu}}_g$:(6 × 4) × 4 bytes = 96 bytes;	Total size of the feature matrix:
	$\tilde{\varSigma}$ : (6 × 4) × (6 × 4) × 4 bytes = 2304 bytes;	(6 4) × 772 × 7 × 4 bytes = 518784 bytes
	Total: 96 × 7 + 2304 = 2976 bytes = 2.9 Kbytes	= 506.6 Kbytes
TR1 subject (772 windows for each class, 5 classes, 6 channels, 4 features per channel)	${\tilde{\mu}}_g$:(6 × 4) × 4 bytes = 96 bytes;	Total size of the feature matrix:
	$\tilde{\varSigma}$ : (6 × 4) × (6 × 4) × 4 bytes =2304 bytes;	(6 × 4) × 772 × 5 × 4 bytes = 370560 bytes
	Total: 96 × 5 + 2304 = 2784 bytes = 2.7 Kbytes	= 361.9 Kbytes

### Real-time performance of the SFTM

**Figure 5:**
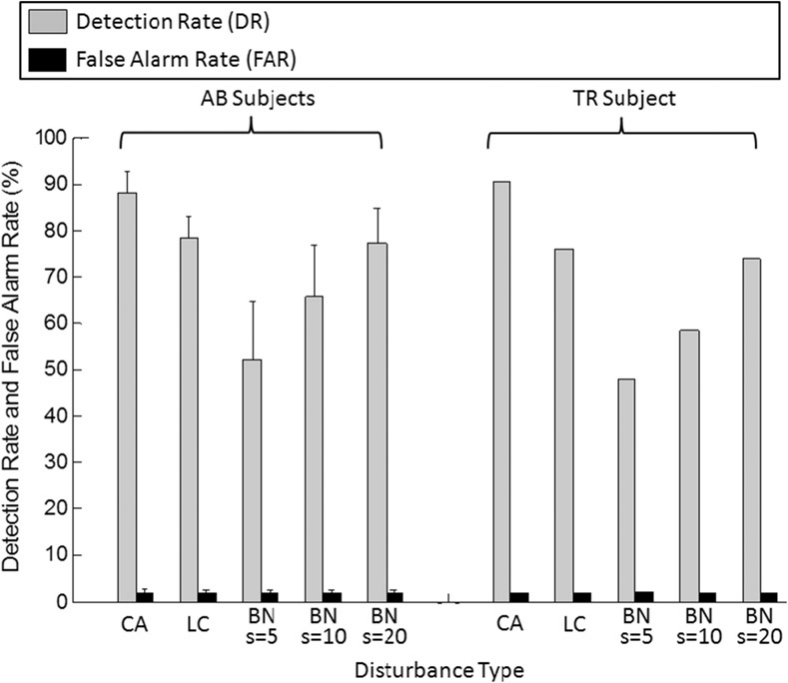
**Detection rate and false alarm rate of the sensor fault detector for different types of disturbances.** For AB subjects, the results were averaged across the five able-bodied subjects. Error bars show ± standard deviation.

**Figure 6:**
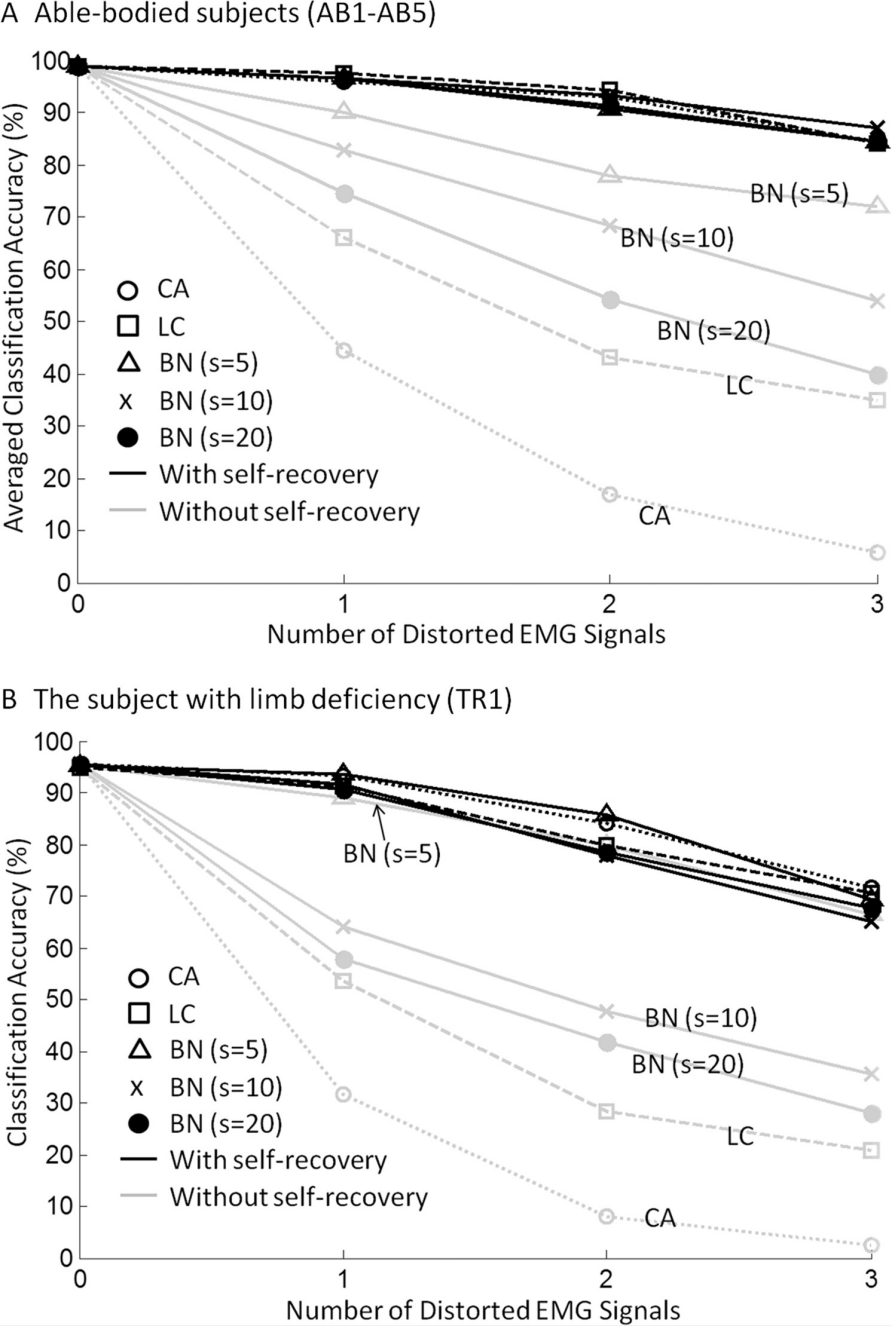
**System classification accuracy with and without the SFTM.** The classification accuracy of the EMG PR system with (black lines) and without (grey lines) the SFTM as the number of distorted signals increased. **(A)**: the classification accuracy averaged across five able-bodied subjects (AB1-AB5). More detailed results showing the values of mean ± standard deviation and the results of statistical analysis are presented in Table 3. **(B)**: the classification accuracy for the subject with limb deficiency (TR1).

Figure 5 shows the detection rate (DR) and the false alarm rate (FAR) of the sensor fault detector for different types of disturbances. Figure 6 plots the motion classification accuracies of the EMG PR system with (black lines) and without (grey lines) the SFTM. More detailed results of the averaged classification accuracy (mean ± standard deviation) across the five able-bodied subjects and the results of statistical analysis are presented in Table 3. As shown in the gray lines in Figure 6, different disturbance types had different damage levels to the system performance. According to the damage level, the five investigated disturbance types can be ranked as CA, LC, BN(*s* = 20), BN(*s* = 10), and BN(*s* = 5), where the CA disturbances caused the largest performance reduction while the BN(*s* = 5) only had slight impact on the classification performance. As shown in Figure 5, the DR of the sensor fault detector increased as the damage level of the disturbance increased. Although the DR had large variation among disturbance types (DR ranged from 52% (BN*s* = 5) to 88% (CA) for AB subjects, and from 48% (BN*s* = 5) to 91% (CA) for TR1), the resulted recovered classification accuracies for different types of disturbances were quite similar. A one-way ANOVA test on the five able-bodied subjects suggested that the classification accuracies derived from the system with the SFTM were not significantly different across different disturbance types and levels (p > 0.08).

**Table 3:** **The classification accuracy of the EMG PR system averaged across five able-bodied subjects (AB1-AB5)**

	**CA**	**LC**	**BN**	**BN**	**BN**
			***s*** **= 5**	***s*** **= 10**	***s*** **= 20**
No disturbance (w/o SFTM)	98.88 ± 0.73	98.84 ± 0.51	98.85 ± 0.53	98.90 ± 0.59	98.88 ± 0.57
No disturbance (w/ SFTM)	98.88 ± 073	98.84 ± 0.51	98.82 ± 0.53	98.90 ± 0.36	98.85 ± 0.57
1 channel disturbed (w/o SFTM)	44.50* ±10.50	66.22* ± 10.24	90.10* ± 2.94	82.93* ± 3.07	74.60* ± 2.38
1 channel disturbed (w/ SFTM)	96.00* ± 3.00	97.44* ± 2.23	96.70* ± 2.94	96.71* ± 1.31	96.58* ± 0.60
2 channels disturbed (w/o SFTM)	16.85* ± 12.74	43.05* ± 11.90	77.95* ± 8.30	68.36* ± 13.50	54.42* ± 12.56
2 channels disturbance (w/ SFTM)	92.81* ± 4.64	94.18* ± 5.22	90.80* ± 8.30	93.38* ± 2.63	91.31* ± 4.33
3 channels disturbed (w/o SFTM)	5.88* ± 5.50	35.06* ± 3.62	72.08* ± 5.17	54.02* ± 7.70	39.72* ±14.10
3 channels disturbed (w/ SFTM)	84.47* ± 6.17	84.20* ± 3.91	87.50* ± 5.17	87.19* ± 8.17	84.37* ± 7.34

*Paired *t*-test denotes significant difference between the classification accuracy of the system with and without the SFTM (p < 0.05).

As shown in Table 3, classification accuracy of the EMG PR system was significantly improved by the SFTM when disturbance occurred (paired *t*-test, p < 0.02 for all cells in Table 3). For both the AB subjects and the TR subject, the classification performance of the system deteriorated as the number of distorted signals increased, whether or not the SFTM was employed. Having more distorted channels means it will be more challenging for the sensor fault detector to identify all the disturbances correctly. Meanwhile, the PR system will lose more information if more distorted channels are removed from the system. However, the performance of the system with the SFTM deteriorated much more slowly than the system without the module. The boosts of classification accuracies generally increased with the number of distorted channels. This indicates that the benefit of the SFTM became more obvious when multiple sensors were disturbed at the same time.

It was observed that the redundant information recorded from able-bodied subjects was more than that recorded from the amputee subject. Although the sensor fault detector provided similar DRs for subject TR1 in comparison with the able-bodied subjects, the recovered classification accuracies for TR1 deteriorated faster than those for the able-bodied subjects as shown in Figure 6. This is because the amount of extractable neural information from EMG signals from the TR patient’s deficient forearm was limited. A similar observation was demonstrated in the previous study [24] in which the redundant information recorded from the gluteal and thigh muscles from five able-bodied subjects was found to be more than that recorded from a subject with a long transfemoral amputation.

The FAR of the sensor fault detector for all subjects ranged from around 1% to 2.5% as shown in Figure 5 and Table 4. Overall, the false alarms had very slight effects on the classification decisions as shown in Table 4, which was expected because in the training phase the TCAD caused by false alarms was set to be a small value of 0.2%. When the SFTM was employed, among all subjects and all types of tests, about 0.5% to 5% of the decisions affected by false alarms resulted in classification errors; meanwhile, around 0% to 1.5% of the decisions affected by false alarms were originally erroneous without the SFTM, and then got corrected after the SFTM was employed. Over 90% of the false alarms had no effect to the system performance.

**Table 4:** **The effects of false alarms on the classification decisions**

**Subject**	**AB1**	**AB2**	**AB3**	**AB4**	**AB5**	**TR1**
FAR (%)	1.17	0.98	1.91	2.48	1.79	2.11
EFAR (%)	3.71	4.86	2.61	0.42	0.84	1.86
CEFAR (%)	0.00	0.69	1.42	0.25	0.21	0.93

FAR = false alarm rate.

EFAR = the ratio between the number of classification errors caused by false alarms and the total number of analysis windows affected by false alarms.

CEFAR = the ratio between the number of classification errors corrected by false alarms and the total number of analysis windows affected by false alarms.

**Figure 7:**
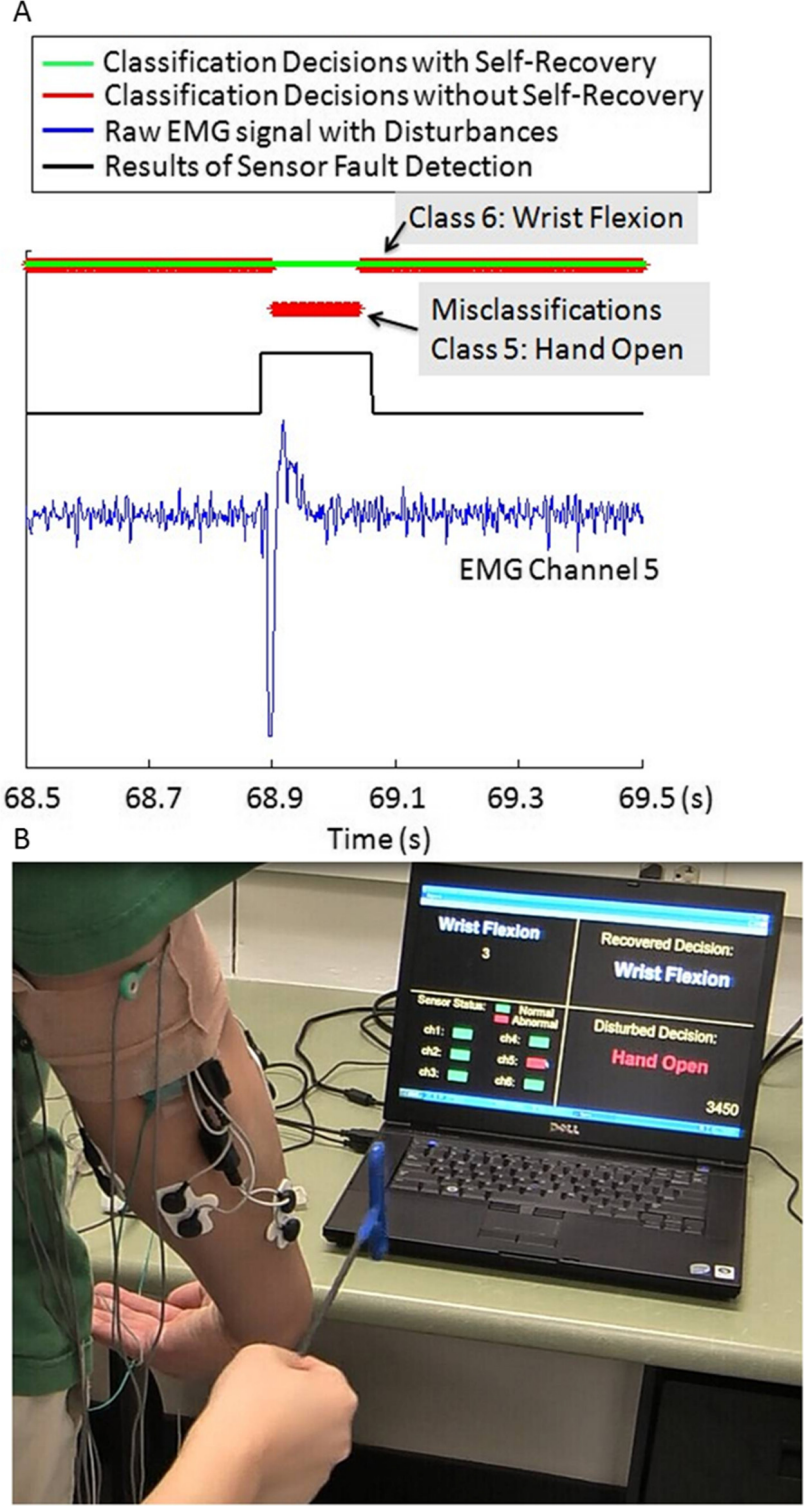
**Real-time performance of the self-recovery system. (A)**: Real-time system performance of a segment of a representative CA trial; **(B)**: A screenshot from the video that recorded the corresponding CA trial presented in **(A)**. The software GUI in **(B)** displayed the performance of the real-time tests. The upper-left panel displayed the instructed motion, which is used to cue the subjects to elicit and relax contractions. The lower-left panel showed the detection result of individual sensor status (green: normal; red: abnormal). The upper-right and the lower-right panels displayed the classification decisions with and without the SFTM, respectively. The number in the lower-right corner was the index of current analysis window in the testing trial.

Figure 7 plots the real-time system performance of a segment of a representative CA trial. In this segment, a CA disturbance was introduced to Channel 5 (extensor pollicis longus) when the subject was performing wrist flexion. The blue line displays the raw EMG signals with the CA disturbance. The black line in the middle represents the results of the sensor fault detection. A high signal means the EMG signal was identified as abnormal, and a low signal means normal. Without the self-recovery module, the wrist flexion motion was misclassified as hand open as displayed in the red line. By employing the SFTM, we can see the disturbance was detected immediately and the classification errors were successfully eliminated in real-time as displayed in the green line. A screenshot from the recorded video of this CA trial is shown in Figure 7. In the screenshot, the experimenter just tapped the electrode of Channel 5. We can see in the software GUI, Channel 5 was identified as abnormal. The classification decisions with and without the SFTM were displayed as wrist flexion and hand open, respectively.

## Discussion

In order to be a useful addition to the existing EMG PR interface for reliable and robust prosthesis control, a practical SFTM has to be fast enough, lightweight, automatic, and most importantly, robust under different conditions with or without disturbances. The experimental results presented in the previous section have demonstrated the feasibility of such an SFTM.

First, the proposed fast LDA retraining algorithm significantly shortened the retraining time from up to 1 s to less than 4 ms when tested on the embedded system prototype, which successfully addressed the most critical challenge in realizing the designed SFTM in real-time. The previous 1 s delay might already generate 50 classification errors (given the analysis window increment of 20 ms) before the SFTM starts to recover the system performance, which is apparently impractical, especially given the fact that some disturbances such as contact artifacts only last for a few hundred milliseconds. That means the disturbance cannot be recovered at all because when the SFTM is ready for recovery, the disturbance is already gone. Shortening the retraining time to less than 4 ms allows the SFTM to complete sensor fault detection, classifier retraining, and pattern classification with the new classifier seamlessly in a sequence within the current analysis window. This clearly demonstrates the feasibility of a nearly “zero-delay” SFTM that is truly imperceptible to users.

Second, the developed SFTM is lightweight in terms of computation and memory overhead, which is a big advantage in applying the SFTM in practice, especially for future embedded system implementation. The fast retraining algorithm and the sensor fault detection algorithm were both smartly designed to efficiently utilize existing information calculated by the EMG PR algorithm, and thus to minimize the computation and memory overhead. The same set of training data used to train the classifier was also used to build the sensor fault detectors so that additional data collection and memory consumption can be avoided.

Third, the outlier threshold of the sensor fault detector was automatically estimated by specifying the value of TCAD in the training phase. This strategy guarantees that an optimized outlier threshold can be determined for most sensitive sensor fault detection under the condition that the SFTM will not significantly harm the EMG PR performance when there is no disturbance. This is also an important factor for applying the SFTM in practice because disturbances do not always exist in the real world. Outlier detection has been a widely used method in anomaly detection for biomedical data [33,36,37], but most existing studies have focused on the detection performance itself. The outlier thresholds are usually determined by specifying the false alarm rate [29]. In our study, we care more about the effect of the selection of outlier threshold on the PR performance rather than that on the detection rate and false alarm rate. Using this threshold tuning strategy, the false alarms generated by the sensor fault detectors only had slight effect on the PR performance, which means the proposed robust EMG PR interface can still maintain its classification performance when there is no disturbance.

The experimental results demonstrated the efficacy of the SFTM for handling the three types of disturbances investigated in this study. Although the sensor fault detection rate had a large variation among different types of disturbances, the recovered classification accuracies derived from testing trials under different conditions were quite similar. This is because low-level disturbances, though difficult to detect, had only small impacts on pattern classification, while high-level disturbances were more easily detected and the potential damage to the system could be effectively eliminated. The benefit of the SFTM generally became more significant as the number of distorted EMG signals increased and as the damage level of disturbance increased. The classification recovery performance for the TR subject deteriorated faster than that for the able-bodied subjects, which indicated that the redundant information recorded from the TR subjects was less than that recorded from the able-bodied subjects. As information redundancy in multiple EMG signals is essential for the success of the SFTM, this might be a challenge to apply the designed SFTM to patients with amputations because they usually have limited EMG recording sites for neural information extraction. As suggested in [24], the advanced surgical technique targeted muscle reinnervation (TMR) [5] is a promising method for solving this problem. The TMR surgery could enhance the information redundancy in recorded EMG signals by transferring the residual nerves that originally commanded the muscles in the missing limb to alternative residual muscles. In addition, in recent years as the MEMS (micro-electro-mechanical systems) technology advances, high-density, micro-electrode EMG arrays have been developed [33,38,39]. These surface EMG arrays allow neural information extraction from both the temporal and the spatial domain, and can provide better-quality neural information than single channel EMG electrodes. Using high-density electrode arrays will result in much more redundant information in the EMG recordings. Furthermore, recording with many electrodes simultaneously often implies disturbances such as bad-contacts [33]. As the high-density EMG arrays become the trend, the SFTM will be more important in the future myoelectric prosthetic technology.

Our proposed SFTM required user-specific training on individual subject for building the motion classifier and the sensor fault detector. In recent years, user-independent motion classification has become a promising approach for eliminating training phase on individual users and has potential for providing more user-friendly and more broadly accessible EMG PR-controlled applications. Ison et al. [40] provides a good review on user-independent EMG PR control approaches. While some recent studies have shown great advances in this field [41-43], there has been no user-independent classification method being validated on amputee subjects in real-time yet. For EMG PR-controlled prostheses, currently user-specific classification is still the dominant approach.

The performance measurement of this study was mainly focused on evaluating the SFTM’s ability of recovering erroneous classifications caused by disturbances and the feasibility of implementing a lightweight, automatic, and robust SFTM in real-time. To apply the SFTM in clinical practice, in recent years, additional functional performance metrics have been considered important to evaluate the usability of real-time PR control systems. Such metrics include completion time, completion percentage, and path efficiency of targeted tasks [5,44,45]. These functional metrics will be evaluated in our future work to investigate how the SFTM will affect the usability of PR control schemes. The three representative disturbances types were chosen for this study because on one hand, they are common and critical disturbances of EMG recordings [10,33], and on the other hand, they are more controllable and relatively easier to simulate and quantify in real-time experiments compared with some other common disturbances such as fatigue, electrode shift, and limb motions. In our future work, more types of practical disturbances will be investigated, including not only more individual disturbance types in the real world such as fatigue, electrode shift, and limb motions, but also coupling of these disturbances according to the realistic situation (e.g. CA and LC often come together). The occurring timing and duration of disturbances is also an important factor for further exploration. Instead of introducing disturbances randomly with random duration, for each type of disturbance, we will study when it typically occurs and how long it usually lasts practically, and consider this information in our experiments. Another future work will be integrating the EMG sensor interface into the embedded prototype of the robust EMG PR interface to be able to sample and digitize EMG signals and conduct real-time tests on a self-contained embedded system. In addition, more amputee subjects will be recruited for further testing of the robust EMG PR interface.

## Conclusions

This paper presented a real-time, practical SFTM for robust EMG PR. A fast LDA retraining algorithm and a fully automatic sensor fault detector based on outlier detection were developed, which allowed the SFTM to promptly detect various disturbances and recover the PR performance with nearly zero delay. In addition, no prior knowledge of disturbance type was required in building the sensor fault detector, and a new method for automatically tuning the outlier threshold in the training phase was developed. The SFTM was skillfully designed to make the most efficient use of existing information calculated by the EMG PR algorithm, and thus added little computation and memory overhead to the EMG PR interface. An integrated working prototype of the SFTM was then built for evaluation of the system validity, sensitivity, reliability, and robustness in real-time. The experimental evaluation on five able-bodied subjects and one TR amputee subject showed that the PR interface with the SFTM could obtain significantly improved classification accuracy in comparison with the interface without the SFTM when different types of disturbances were introduced. These results have demonstrated the feasibility of a clinically viable and robust EMG PR interface for multifunctional prosthetic arm control.

## Abbreviations

PR: Pattern recognition
SFTM: Sensor fault-tolerant module
LDA: Linear discriminant analysis
TD: Time-domain
TCAD: Tolerable classification accuracy degradation
TR: Transradial
CA: Contact artifact
LC: Loose contact
BN: Baseline noise
COM: Computer-on-module
DR: Detection rate
FAR: False alarm rate
TMR: Targeted muscle reinnervation
